# Interpreting Clinical Trials With Omega-3 Supplements in the Context of Ancestry and *FADS* Genetic Variation

**DOI:** 10.3389/fnut.2021.808054

**Published:** 2022-02-08

**Authors:** Floyd H. Chilton, Ani Manichaikul, Chaojie Yang, Timothy D. O'Connor, Laurel M. Johnstone, Sarah Blomquist, Susan M. Schembre, Susan Sergeant, Manja Zec, Michael Y. Tsai, Stephen S. Rich, Susan J. Bridgewater, Rasika A. Mathias, Brian Hallmark

**Affiliations:** ^1^School of Nutritional Sciences and Wellness, University of Arizona, Tucson, AZ, United States; ^2^BIO5 Institute, University of Arizona, Tucson, AZ, United States; ^3^Center for Public Health Genomics, University of Virginia, Charlottesville, VA, United States; ^4^Department of Biochemistry and Molecular Genetics, University of Virginia, Charlottesville, VA, United States; ^5^Program in Personalized and Genomic Medicine, Department of Medicine, Institute for Genome Sciences, University of Maryland School of Medicine, Baltimore, MD, United States; ^6^University of Arizona Genetics Core, University of Arizona, Tucson, AZ, United States; ^7^Department of Family and Community Medicine, College of Medicine-Tucson, University of Arizona, Tucson, AZ, United States; ^8^Department of Biochemistry, Wake Forest School of Medicine, Winston-Salem, NC, United States; ^9^Department of Laboratory Medicine and Pathology, University of Minnesota, Minneapolis, MN, United States; ^10^College of Medicine, University of Arizona, Tucson, AZ, United States; ^11^Department of Medicine, Johns Hopkins University, Baltimore, MD, United States; ^12^Center for Biomedical Informatics and Biostatistics, BIO5 Institute, University of Arizona, Tucson, AZ, United States

**Keywords:** polyunsaturated fatty acid, fatty acid desaturase, gene-diet interaction, oxylipins, endocannabinoid, omega-3 supplements, ancestry, omega-3 deficiency syndrome

## Abstract

Human diets in developed countries such as the US have changed dramatically over the past 75 years, leading to increased obesity, inflammation, and cardiometabolic dysfunction. Evidence over the past decade indicates that the interaction of genetic variation with changes in the intake of 18-carbon essential dietary omega-6 (n-6) and omega-3 (n-3) polyunsaturated fatty acids (PUFA), linoleic acid (LA) and α-linolenic acid (ALA), respectively, has impacted numerous molecular and clinical phenotypes. Interactions are particularly relevant with the *FADS1* and *FADS2* genes, which encode key fatty acid desaturases in the pathway that converts LA and ALA to their long chain (≥20 carbons), highly unsaturated fatty acid (HUFA) counterparts. These gene by nutrient interactions affect the levels and balance of n-6 and n-3 HUFA that in turn are converted to a wide array of lipids with signaling roles, including eicosanoids, docosanoids, other oxylipins and endocannabinoids. With few exceptions, n-6 HUFA are precursors of pro-inflammatory/pro-thrombotic signaling lipids, and n-3 HUFA are generally anti-inflammatory/anti-thrombotic. We and others have demonstrated that African ancestry populations have much higher frequencies (vs. European-, Asian- or indigenous Americas-ancestry populations) of a *FADS* “derived” haplotype that is associated with the efficient conversion of high levels of dietary n-6 PUFA to pro-inflammatory n-6 HUFA. By contrast, an “ancestral” haplotype, carrying alleles associated with a limited capacity to synthesize HUFA, which can lead to n-3 HUFA deficiency, is found at high frequency in certain Hispanic populations and is nearly fixed in several indigenous populations from the Americas. Based on these observations, a focused secondary subgroup analysis of the VITAL n-3 HUFA supplementation trial stratifying the data based on self-reported ancestry revealed that African Americans may benefit from n-3 HUFA supplementation, and both ancestry and *FADS* variability should be factored into future clinical trials design.

## Background

Highly unsaturated fatty acids (HUFA)—polyunsaturated fatty acids (PUFA) with ≥20 carbons and ≥3 double bonds—and signaling metabolites derived from them play key roles in inflammation and thrombosis that contribute to numerous medical conditions including cardiovascular disease (CVD), Alzheimer's disease (AD), type 2 diabetes, autoimmunity, cancer, hypersensitivity disorders, skin and digestive disorders, and infectious disease such as COVID-19 (1, 2). More specifically, the ratios of circulating and cellular levels of omega-3 (n-3) and omega-6 (n-6) HUFA dictate the balance of inflammatory and thrombotic signaling molecules such as eicosanoids and other oxylipins. The proportions of oxylipins, such as eicosanoids and docosanoids, modulate a wide variety of physiological and pathophysiological functions *via* their capacity to mediate numerous biological processes including inflammation and blood clotting (3–5).

**Figure 1:**
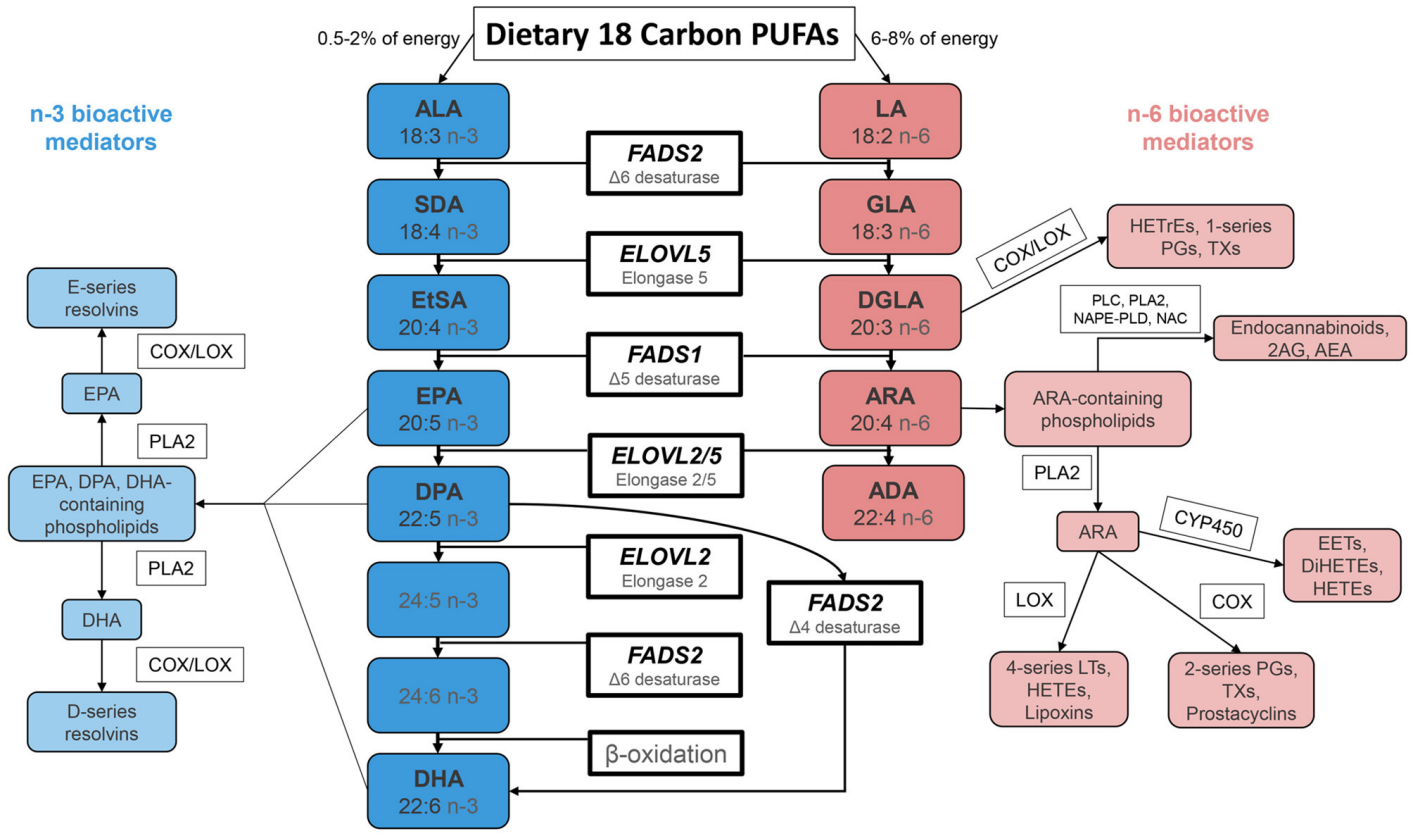
Biosynthesis of n-3 and n-6 Highly Unsaturated Fatty Acids and Lipid Signaling Products. N-6 and n-3 HUFA are synthesized from dietary intake of essential fatty acids ALA and LA, respectively, through a series of enzymatic desaturation (FADS2 and FADS1) and elongation (ELOVL2 and ELOVL5) steps. This pathway gives rise to primary n-3 HUFA and n-6 HUFA such as EPA, DPA, DHA and ARA. These LC-PUFA (as free fatty acids or complex lipids) and lipid signaling metabolites impact a wide range of physiologic and pathophysiologic processes. FADS 1/2, fatty acid desaturase 1/2; ELOVL 2/5, fatty acid elongase 2/5; ALA, α-linolenic acid; SDA, stearidonic acid; EtSA, eicosatetraenoic acid; EPA, eicosapentaenoic acid; DPA, docosapentaenoic acid; DHA, docosahexaenoic acid; LA, linoleic acid; GLA, γ-linolenic acid; DGLA, dihomo-γ-linolenic acid; ARA, arachidonic acid; ADA, adrenic acid; PG, prostaglandin; TX, thromboxane; LT, leukotriene; HEPE, hydroxyeicosapentaenoic acid; HETrE, hydroxyeicosatrienoic acid, HETE, hydroxyeicosatetraenoic acid; DiHETE, dihydroxyeicosatetraenoic acid; EET, epoxyeicosatetraenoic acid; 2AG, 2-arachidonoylglycerol; AEA, arachidonoyl ethanolamide/anandamide.

The 18-carbon n-3 and n-6 dietary PUFA, alpha linolenic acid (ALA) and linolenic acid (LA) are essential nutrients throughout the animal kingdom. ALA is generated in plants utilizing a methyl-end desaturase Δ-15 desaturase (ω3 desaturase) that adds a double bond to LA between the n-6 double bond and the methyl end of the hydrocarbon chain (6). Once formed and ingested by humans, ALA can be converted to n-3 HUFA, including eicosapentaenoic acid (EPA), docosapentaenoic acid (DPA), and docosahexaenoic acid (DHA), utilizing several desaturation and elongation steps (Figure 1) (2). Similarly, a second series of n-6 HUFA including dihomo gamma linolenic acid (DGLA), arachidonic acid (ARA), and adrenic acid (ADA) can be formed by the same biosynthetic pathway and compete with the corresponding n-3 species for the same enzymes. With few exceptions, ARA is a precursor to pro-inflammatory/pro-thrombotic signaling molecules, and n-3 HUFA are generally metabolized to anti-inflammatory/anti-thrombotic products (3–5).

In 1961, the American Heart Association recommended that dietary PUFA be substituted for saturated fats “as a possible means of preventing atherosclerosis and decreasing the risk of heart attacks and strokes” (7). This recommendation was largely based on data showing that this substitution of predominately the n-6 PUFA, LA, lowered serum total and LDL cholesterol (8, 9). As a result, increased ingestion of LA-containing vegetable oils and processed foods has increased dietary LA dramatically (to 6–9% of daily energy) and has increased the ratio of dietary n-6/n-3 PUFA to >10:1 (10, 11). Concerns have arisen over the rapid increases in dietary LA levels and the resulting imbalance in n-6 to n-3 PUFA, and intense controversy remains over the health and disease implications of these levels of dietary LA (11–20).

For example, studies are consistent that replacing saturated fats with vegetable oils high in LA reduces serum cholesterol. Further, the traditional diet-heart hypothesis would predict that this would lead to lower deposition of cholesterol in arterial walls, attenuation of atherosclerosis resulting in reduced coronary artery events and all-cause mortality. However, not all studies show this replacement reduces coronary heart disease and all-cause deaths (12, 13, 20, 21). In fact, Ramsden et al. demonstrated in analysis of data from the Sidney Diet Heart Study (a single blinded, parallel group, randomized controlled trial, *n* = 458) that selectively increasing LA (to ~15% of food energy) from safflower oils and safflower margarine increased risk of cardiovascular risk and all cause mortality by 35% and 29%, respectively, compared to diets enriched in saturated fats (to ~10% of food energy) (20). Similar results were observed in the Minnesota Coronary Experiment, a double blind randomized controlled trial designed to test replacement of saturated fat with vegetable oil rich in linoleic acid (13). In contrast, multivariable-adjusted associations of circulating or adipose tissue LA demonstrate that higher levels of LA (expressed as % of total fatty acids) are significantly associated with lower risks of cardiovascular mortality and all-cause mortality (22, 23).

Collectively, these studies have raised important questions as to why these results are so inconsistent especially as it relates to such a vital health question. In a meta-analysis of randomized clinical trials (RCTs), Ramsden et al. showed that mixed n-3 ALA/n-6 LA vs. n-6 specific LA interventions have significantly different effects on coronary heart disease (CHD) risk with mixed interventions reducing the risk of non-fatal myocardial infarction (MI) and non-fatal MI+CHD death, while specific LA diets increased risk of all coronary heart disease endpoints (12). We have demonstrated that the method used to express levels of PUFA and HUFA can affect the magnitude and direction of associations with blood lipids (24), and it remains to be seen whether this impacts CHD endpoints or mortality risk. Importantly for the purpose of this review, there are no studies on the impact of dietary LA in the context of ancestry, which is a critical missing gap given our knowledge of the role of ancestry-driven genetic variation in HUFA metabolism summarized below.

Up until a century ago, LA in the diet was limited (2, 25–28). Human diets were largely limited to plants, naturally grazing animals, and fish, which all had much lower levels of LA and an overall dietary ratio of LA to ALA of ~2:1, as opposed to the >10:1 observed today (10). Available data suggests that dietary LA provided 2 to 3 % of daily energy at most until the late nineteenth and twentieth centuries (27, 29). Additionally, up until that time, the diet contained much more balanced levels of n-3 and n-6 HUFA. LA was dramatically increased in the food supply of western countries as the result of several technological events and dietary recommendations (2). There was the commercial refining of LA-rich seed oils, the invention of hydrogenation that enabled seed oils to be solidified in shortenings and margarines, and the substitution of animal fats with LA enriched oils. These taken together with several dietary recommendations that PUFA (particularly LA) be substituted for saturated fats (8, 9) dramatically increased LA levels an estimated four-fold and markedly increased ratios of LA to ALA and n-6 to n-3 HUFA by reducing n-3 HUFA by an estimated 40% (10).

The effects of this increase in LA have been debated, with one systematic review finding that large changes in LA intake resulted in little to no changes in tissue ARA levels (30). However, given the shared enzymatic steps involved in the metabolism of LA and ALA (Figure 1), these PUFA and their intermediates compete in the liver and other tissues for enzymatic reactions that produce biologically active HUFA. Consequently, a marked increase in LA reciprocally deceases levels of all major n-3 HUFA including EPA, DPA and DHA (31–35), while a reduction in LA increases these n-3 HUFA. Additionally, the conversion of PUFA to HUFA reaches a saturation point at which additional PUFA have no effect on HUFA levels (36). Recent studies from our lab reveal that this saturation occurs at the *FADS1* (Δ-5 desaturase) step in the biosynthetic pathway (37–39) and flux through this step is markedly altered by genetics as described in detail below.

Another concern with increased ratios of LA to ALA is related to oxylipins (including eicosanoids and docosanoids) and other signaling molecules that are synthesized from n-6 and n-3 HUFA. ARA-derived eicosanoids compared to EPA and DHA-derived eicosanoids, docosanoids, and specialized pro-resolving lipid mediators typically have opposing biological effects (3–5), and a precise HUFA balance is therefore critical to avoid hyperinflammatory and hypercoagulopathy events in numerous human conditions (1). While the effects of increasing tissue ARA are not completely clear (40), the importance of this balance was demonstrated 20 years ago when selective cyclooxygenase 2 inhibitors were removed from the market after an increase in the number of thrombotic events. This was attributed to an enhanced production of thromboxane A2 *via* the cyclooxygenase 1 enzyme coupled with a reduction in levels of prostacyclin (41). This lack of a “balanced HUFA milieu” has also recently been proposed to be important for the avoidance of the cytokine storm and excess clotting associated with COVID-19 (1). However, it is important to point out that there are a large number of oxylipins derived from n-6 and n-3 PUFA and HUFA that can be produced enzymatically and by autooxidation, and this results in an immense complexity of physiological effects that are not yet fully understood (42).

In summary, the production and human consumption of LA has risen dramatically over the past 100 years (2), and LA now represents over 90% of total PUFA + HUFA intake in Western diets (10). Nutrient deficiencies or imbalances have historically occurred as a result of inadequate food consumption; however, in this case, the amount of PUFA entering the HUFA biosynthetic pathway has been immensely changed by manipulation of food supplies, increases in processed foods, and dietary recommendations. Moreover, in countries such as the US, LA-containing food and oils are being consumed by highly diverse populations representing numerous ancestries and evolutionary histories. The purpose of this review is first to describe the potential impact of PUFA-based gene-diet interactions that lead to ancestry- and gene variation imbalances in n-6 HUFA to n-3 HUFA levels. Second, we will provide a rationale for how these can provide metabolic underpinnings for much of the observed clinical trial heterogeneity with n-3 HUFA supplementation trials. Finally, we describe how precise dietary and supplementation strategies with n-3 HUFA could rebalance HUFA to prevent and manage human diseases especially in certain human populations.

## The Biochemistry and Genetics of n-6 and n-3 HUFA Formation

Early studies carried out in European ancestry populations showed that humans had the capacity to convert only a small proportion (3–4% of calories/kilojoules) of ingested LA and ALA into n-6 and n-3 HUFA (36, 43). It was also recognized that this was largely due to the desaturase steps (Δ6 and Δ5), encoded by two genes (*FADS2* and *FADS1*) in the *FADS* cluster on chromosome 11 (chr11q12-13.1) (44). Based on these assumptions, theoretically, recommendations to increase dietary LA to 6–8% of energy should not fundamentally impact HUFA levels, because at most, only 4% of LA could be converted to ARA. Thus, the impact of high dietary LA on ARA signaling products such as oxylipins (including eicosanoids) and endocannabinoids was thought to be limited by the efficiency of conversion of LA to ARA, and this was assumed to be similar across human populations.

Around 2006, candidate gene studies and genome-wide association studies (GWAS) began to show that genetic variation in the fatty acid desaturase cluster, which includes *FADS1, FADS2*, and *FADS3*, and also in the fatty acid elongase genes, which include *ELOVL5* and *ELOVL2*, was highly associated with tissue and circulating levels of PUFA and HUFA (45–52). These initial studies, conducted mostly in European-ancestry individuals, revealed variability in the genes associated with HUFA biosynthesis and also demonstrated that this genetic variability impacted the efficiency of the HUFA biosynthetic pathway. Importantly the same variants found in HUFA association studies were also reported in relation to several human diseases including CVD, metabolic syndrome, obesity, atopic dermatitis, and major depressive disorders (1, 44, 53, 54).

In particular, an early study by Martinelli et al. showed that a higher ratio of ARA to LA in individuals with the *FADS* haplotype for efficient conversion of LA to ARA was an independent risk factor for coronary artery disease (55). Additionally, levels of high-sensitivity c-reactive protein, which is an inflammatory marker associated with risk of CVD, increased progressively with the haplotype-dependent ratio of AA to LA. The authors concluded that in a Western diet, “subjects carrying *FADS* haplotypes that are associated with higher desaturase activity may be prone to a proinflammatory response favoring atherosclerotic vascular damage.” This study and similar ones began to raise the alarm that “one size fits all” recommendations related to high dietary LA may actually harm certain individuals in a highly diverse population such as the US.

Recently, in a study that combined metabolomic and GWAS analyses, we examined the capacity of *FADS* variants to impact the balance of pro- vs. anti-inflammatory or thrombotic HUFA metabolite balance (38). This study was designed to examine not only the rate-limiting step of HUFA biosynthesis but also regions of the genome that exert genetic control over a large number the HUFA-containing complex lipids and signaling molecules. We examined associations between levels of 247 lipid metabolites (including four major classes of HUFA-containing molecules and signaling molecules) and common and low-frequency genetic variants throughout the genome. Genetic variation within the *FADS* locus was strongly associated with 52 HUFA-containing lipids and signaling molecules, including free fatty acids, phospholipids, lyso-phospholipids, and an endocannabinoid (2-arachidonyl glycerol). The HUFA within these lipids were largely a precursor (such as dihomo gammalinolenic acid [DGLA]) or a product (such as ARA) of the Δ5 desaturase (*FADS1*) step. Perhaps most surprisingly, for over 80% of the *FADS*-associated lipids, there were no significant genetic associations outside the *FADS* locus, offering further evidence of the impact of this single genetic locus. This was unanticipated as many of these HUFA-containing lipids were synthesized by up to five biochemical steps past the *FADS1* desaturation step. These data suggest that *FADS* variation is the critical “control point” in the formation and levels of numerous biologically important, HUFA-containing lipids and signaling molecules, many of which are related to health and human disease outcomes.

Given the role of the *FADS1* step as a central control point of so many biologically important lipids, it is important to also consider the effect of genetic variation on the precursor-product flux across the *FADS1* desaturation step. In particular, it was important to understand the effect of genetic variation on levels of the precursor of the *FADS1* desaturation step, DGLA, which is converted to anti-inflammatory, anti-thrombotic, and vasodilatory signaling molecules and the product of this step, ARA, which is the precursor to largely pro-inflammatory, pro-thrombotic, and vasoconstricting products. Kothapalli et al. recently modeled the DGLA to ARA flux across the *FADS1* step to be altered by 84% between the DD and II genotype of the *FADS* insertion/deletion variant (indel) rs66698963 (1, 56). We found a similar impact on flux of 82% measuring DGLA and ARA levels when comparing the GG and the TT genotypes for the variant rs174537 (37) near *FADS1/2*. When this relationship is further modeled using LA quantities in Western diets, not only does the ratio of ARA to DGLA shift dramatically between genotypes but the ratio of ARA to the sum of all n-3 HUFA is projected to increase by 47% (1). As highlighted in the section on ancestry below, this becomes highly relevant when population frequencies differ dramatically, and haplotypes in many populations are fixed with almost all individuals harboring the higher flux genotypes.

The importance of circulating and red blood cell levels of n-3 HUFA to human health has been demonstrated in several studies and meta-analyses (57–59). In general, these studies show that higher levels of n-3 HUFA are associated with lower risk of coronary heart disease and all-cause mortality. A recent meta-analysis from 17 prospective studies with a median of 16 years of follow up in 42,466 individuals showed a 15–18% reduction in all-cause mortality when comparing the highest and lowest quintiles (57). An important limitation of these studies is they have not included ancestry-based subgroup analyses that, based on the discussion below, are likely to be very important.

The degree to which such HUFA imbalances affect human disease is unknown, but these studies certainly raise concerns as to whether interactions between the homozygous efficient converter, *FADS* genotypes and current levels of LA in Western diets place certain individuals at greater risk for disease due to elevated n-6 HUFA levels and n-6 HUFA to n-3 HUFA ratios. The above-mentioned analyses also raise the question of whether stratifying individuals by *FADS* genotypes/haplotypes may represent an important opportunity for rebalancing n-6 to n-3 HUFA ratios with n-3 HUFA supplementation. We provide evidence to support this hypothesis in the last section of this paper.

## Impact of Ancestry and Associated *FADS* Genetic Variation on HUFA Levels and Human Disease

### African Ancestry

In 2012, it was discovered by our group that African American (AfAm) and European American (EuAm) populations in the US had dramatic differences in both levels of HUFA and the frequency of more efficient *FADS* variants (60, 61). It was also reported that the substantially higher levels of HUFA found in AfAm vs. the EuAm could be explained in part by significant differences in frequency of *FADS* alleles associated with efficient HUFA biosynthesis in the two populations; notably the effect sizes were similar across populations but the frequencies were not. More specifically, only ~45% of EuAm were homozygous for the efficient allele for the variant rs174537 vs. ~80% of AfAm. At about the same time, Ameur et al. identified a set of 28 SNPs in a primary haplotype block that clearly distinguished what was called the “ancestral” and “derived” haplotypes with the derived haplotype having enhanced HUFA biosynthetic capacity (62). Evidence was then provided that these pathway efficient alleles that reside on the derived haplotype in the *FADS* gene cluster were driven to fixation on the African continent ~85 thousand years ago (kya) by positive selection (63). This in turn would have facilitated the production of circulating and tissue HUFA from plant-based PUFA. As HUFA are critical to brain development/function and innate immunity, this study proposed that the selection event played a key role in “allowing African populations obligatorily tethered to marine sources for HUFA in isolated geographic regions, to rapidly expand throughout the African continent 60–80 kya” (63). It also explained the high frequency of pathway efficient alleles in African ancestry populations.

**Figure 2:**
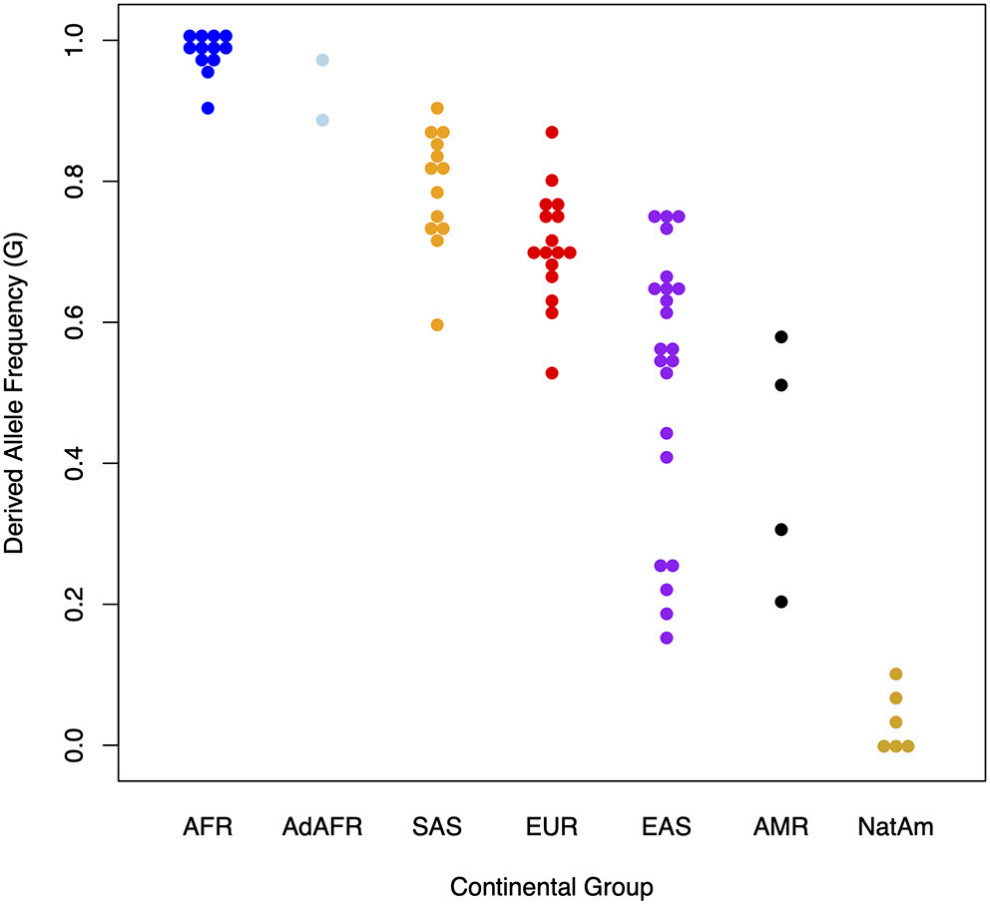
Presence and absence of the derived allele (G) at rs174537 in 80 globally diverse populations. The *Y* axis illustrates the proportion of the derived allele (G) at rs174537 and the X axis shows global populations. AFR, African; AdAFR, Ad Mixed African; SAS, South Asian; EUR, European; EAS, East Asian; AMR, Ad Mixed American; NatAm, Native American.

Figure 2 illustrates the dramatic differences in the frequencies of the derived allele at rs174537 in modern global populations. Looking at 80 globally diverse populations separated broadly by continent of origin, we find a near absence of the ancestral allele in Africa, with various levels of polymorphisms elsewhere. The other exception to this is in the Americas, where we have shown previously an absence of the derived haplotype in many populations (64). Overall, this demonstrates a pattern of repeated natural selection on both the ancestral and derived variants at different times and places across the globe (65).

With regard to African ancestry populations, data from the studies raised questions as to the impact of a Western diet (containing 6–8% LA and a LA to ALA ratio >10:1) on these populations, which have a high frequency of alleles associated with efficient HUFA biosynthesis. Both observational genetic association studies suggesting potential interactions (genetic allele frequencies do not completely explain population differences), and recent clinical trials (offering empirical evidence of gene-nutrient interactions) suggest that this gene-diet interaction would lead to a marked increase in the production of ARA relative to DGLA as well as reducing n-3 HUFA including EPA, DPA and DHA by about 50% in AfAm and other African-ancestry populations (1, 37, 66, 67).

**Figure 3:**
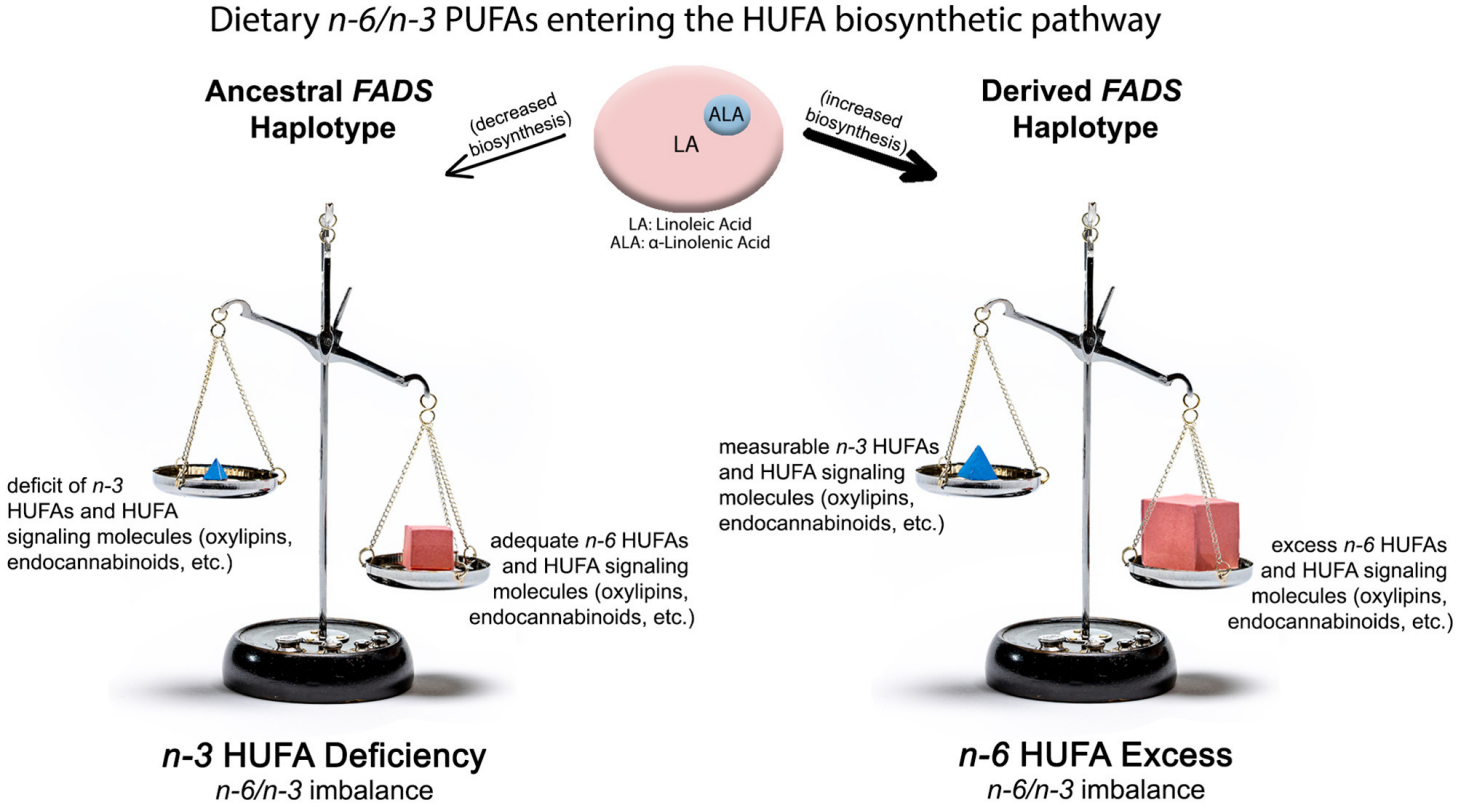
Impact of ancestral and derived *FADS* haplotypes combined with n-6 and n-3 PUFA from western diets on producing n-3 HUFA deficient (Left Side) and n-6 HUFA excess (Right Side) molecular phenotypes. The top of the illustration shows quantities and ratios of dietary PUFA entering the lower efficiency “ancestral” pathway or the higher efficiency “derived” pathway. The lower shows n-6 and n-3 HUFA levels, metabolites and imbalances produced by these gene-diet interactions.

Figure 3 shows the relationship between n-6 and n-3 HUFA levels and global proportions of African and Amerind ancestry in AfAm and Hispanic American participants from the Multi-Ethnic Study of Atherosclerosis (MESA) cohort. Figure 3E illustrates the impact of African ancestry on ARA levels in circulating phospholipids. Total ARA levels (expressed as % of total fatty acids) increased as a function of African ancestry by ~30%. Figures 3A,C show that n-3 HUFA, EPA and DHA also increased by ~30 and ~50%, respectively. These data reveal the impact of African ancestry in AfAm MESA participants on n-6 and n-3 HUFA levels. Figures 3B,D,E (described in detail below) show the inverse impact of Amerind (AI) ancestry on HUFA levels in circulating phospholipids.

Importantly, as discussed above, combined genetic and metabolomic analyses reveal that this shift is not only seen in the HUFA themselves but also HUFA-containing complex lipids and signaling molecules. Additionally, a separate study revealed an association between *FADS* genotype and the ratio ARA/DGLA as well as the biosynthesis of 5-lipoxygenase products produced in whole blood (68). Collectively, these studies show that elevated levels of LA combined with *FADS (*particularly *FADS1)* genetic variability create different mixtures of HUFA that serve as the precursors of critical signaling lipids (oxylipins including eicosanoids and endocannabinoids). These data would lead to the conclusion that African ancestry populations consuming a Western diet with high LA could be impacted more by gene-diet interactions that lead to a HUFA balance predicted to move toward proinflammatory and prothrombotic signaling molecules potentially contributing to disease severity and disease disparities.

**Figure 4:**
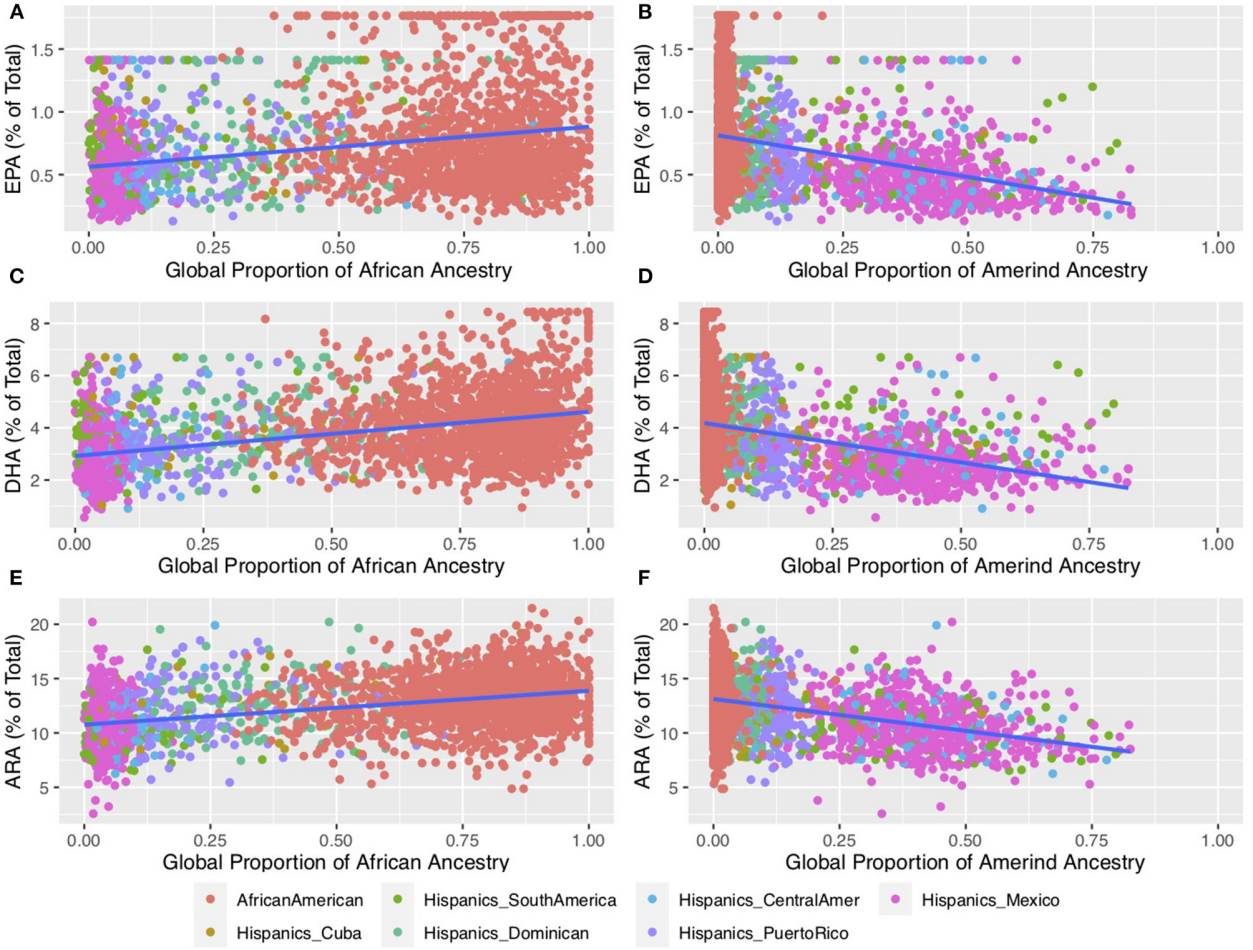
Impact of African and Amerind ancestry on n-6 and n-3 HUFA Biosynthesis. This plot shows HUFA levels for self-reported African American (*n* = 1,505) and Hispanic (*n* = 1,102) participants from MESA (69). Fatty acids were measured using the methods described in Cao et al. (70) and outliers were winsorized to the value of median +/– 3.5 * median absolute deviation (MAD) (39). Proportions of African and Amerind ancestry were estimated using ADMIXTURE (71) using selected Amerind (39) and all African reference samples from the 1,000 Genomes (72) and the HGDP6 (73, 74). **(A,C,E)** illustrate circulating phospholipid levels containing EPA, DHA, and ARA, respectively, as a function of African Ancestry. **(B,D,F)** illustrate circulating phospholipid levels containing EPA, DHA, and ARA, respectively, as a function of Amerind Ancestry. EPA, eicosapentaenoic acid; DHA, docosahexaenoic acid; ARA, arachidonic acid.

Figure 4 (Right Side) illustrates the potential impact of two alleles of derived, pathway efficient variants combined with current levels of LA on levels of n-6 and n-3 HUFA. Here, the biosynthetic efficiency through the FADS (and particularly through the FADS1 [D5 desaturase]) enzymatic step is maximized and produces high, excess levels of n-6 HUFA as well as their signaling metabolites. Under these same circumstances, n-3 HUFA levels and signaling products are measurable but not sufficient to balance the excess quantities of n-6 HUFA synthesized from high quantities of dietary LA entering the highly efficient pathway. With *FADS* variants associated with the derived haplotype, the n-6 HUFA to n-3 HUFA ratio more closely reflects the >10:1 LA/ALA entering the pathway. The mixture of n-6 HUFA to n-3 HUFA serving as substrates for the biosynthesis of signaling molecules is critical as these are typically competitive enzymatic reactions.

### European and Asian Ancestry

Approximately 44% of the European ancestry population in the US have two *FADS* alleles associated with the derived haplotype (60, 61). By comparing *FADS* sequencing data from present-day and Bronze Age (5–3k years ago), Mathieson et al. and Buckley et.al showed that selective patterns observed in Europeans were likely driven by a change in the dietary composition of PUFA following the transition to agriculture (75, 76). This transition gave rise to high intake of LA and ALA and lower ingestion of HUFA thus driving the need for more efficient biosynthesis of HUFA. Figure 2 shows a great deal of variability in the frequency of ancestral and derived variants in Asian populations, with the derived haplotype proportion ranging from ~0.4 in east Asian to ~0.8 in south Asian to populations. In south Asia, Kothapalli et al. showed positive selection for an insertion-deletion mutation (rs66698963) in *FADS2* leading to more efficient biosynthesis of HUFA and proposed this too may have been an adaptation to a more vegetarian diet (56).

### Amerind Ancestry

In contrast to African ancestry populations, in AI-ancestry Hispanic populations the ancestral haplotype that harbors *FADS* variants associated with limited biosynthetic efficiency is found at high frequency and the alleles are nearly fixed in many Native American populations (Figure 2). Fumagalli et al. first found strong signals for the ancestral haplotype in the *FADS* cluster when examining natural selection to cold adaptations in an indigenous Greenland Inuit population (77). The identified variants were also strongly associated with body weight and height, and these findings were replicated in European populations. We confirmed that the ancestral haplotype is fixed in Indigenous Americans in Peru and provided evidence that positive selection possibly continued after the founding of the Americas (64). Our study also demonstrated that the ancestral haplotype is at higher proportions in more northern regions of Siberia, independent of European admixture. Mathieson et al. suggest that the current distribution of the haplotype indicates that Indigenous Americans retained it from Paleolithic Eurasians (65).

The positive selection toward the ancestral haplotype is puzzling given the potential detrimental health effects of a reduced capacity to synthesize HUFA given their role in brain function/development and innate immunity. However, HUFA and particularly n-3 HUFA from cold water marine sources would have likely been the major food source for early Siberian and indigenous American ancestors that remained isolated, possibly in Beringia (78). There is evidence that the ancestral haplotype provided a cold adaptation advantage, but it is not apparent how. As discussed above, it is worth noting that a recent combined genetics and metabolomics study has shown that the *FADS* locus (mainly *FADS1*) is a central control point for signaling lipids such as endocannabinoids, and endocannabinoids are known to impact anthropomorphic and other phenotypic characteristics that could have been critical to cold adaptation (38, 79).

These genetic observations raise questions as to the biochemical and clinical ramifications of the ancestral haplotype for modern populations and particularly those that find themselves ingesting Western diets. In 1997, Okuyama and colleagues made a compelling case that excess LA, generating a dramatic increase in the dietary LA to ALA ratio, would lead to n-3 HUFA deficiency in certain populations and this in turn would increase the risk of CVD, western-type cancers, cerebrovascular diseases, and mental health disorders (80). Regarding our current understanding of the impact of *FADS* haplotype variants, populations with high frequencies of two *FADS* alleles associated with the ancestral haplotype would be expected to have a limited capacity to synthesize HUFA. A gene-diet interaction then would be predicted to produce low (perhaps deficient) levels of n-3 HUFA given a ratio of LA to ALA of 10:1 and restricted flux through the pathway.

We recently tested this hypothesis in Hispanic American participants originating from Central America, South America, Mexico, Dominican Republic, Cuba, and Puerto Rico in the US-based MESA cohort (39). Not surprising, global proportions of genetic ancestry differed markedly, with Central American, South American, and Mexican populations having high AI ancestry compared to those of Dominican, Cuban, or Puerto Rican origin (which have higher African and European ancestry). This was mirrored by a higher frequency of the ancestral haplotype *FADS* alleles in high AI populations vs. low AI populations. Populations from Central America, South America, and Mexico had ancestral allele frequencies ranging from 0.56 to 0.59, while populations from the Dominican Republic, Cuba and Puerto Rico had ancestral allele frequencies ranging from 0.27 to 0.40. The primary hypothesis of this study was that the levels and ratios of LA and ALA found in western diets would be metabolized through a less efficient pathway in a high proportion of AI individuals due to the elevated frequency of two alleles associated with the ancestral haplotype. This in turn would be proposed to produce adequate levels of ARA, but because of the competition between LA and ALA and their intermediates under conditions of limited metabolism, low (inadequate) levels of n-3 HUFA (EPA, DPA and DHA) would be expected as shown in Figure 4 (Left Side). Without adequate n-3 HUFA and in the presence of the imbalance of n-6 to n-3 HUFA, the anti-inflammatory and lipid lowering functions needed to protect against obesity, CVD, and cardiometabolic disease would be reduced. Such data would once again reveal that “one size fits all” dietary interventions are unlikely to be clinically effective for all populations, given the dramatic differences in *FADS* variant frequencies that impact circulating and tissue levels of n-3 HUFA.

Figures 3B,D,F shows that in MESA, AI ancestry was associated with lower levels of n-6 and n-3 HUFA in Hispanic individuals (39). When comparing participants with the highest AI ancestry to those with the lowest AI ancestry, there was a 60, 47 and 31% reduction in EPA, DHA and ARA, respectively, in circulating phospholipids. These declines gave rise to predicted levels among individuals with 100% AI ancestry of ~0.3 and ~2% of total fatty acids for EPA and DHA, respectively, compared to ~8.6 % for ARA. While it is not possible to determine the levels n-3 HUFA where a deficiency with pathophysiologic impact would occur, these are quantitatively very low concentrations of n-3 HUFA.

The observed inverse relations with AI ancestry and n-3 HUFA levels are to be expected given that the substrate saturation point in the pathway is reduced due to genetic variation associated with the ancestral haplotype. Together, these data reveal that combinations of LA to ALA found in Western diets combined with carrying two alleles associated with the ancestral haplotype and low consumption of preformed dietary n-3 HUFA have the capacity to give rise to Omega-3 Deficiency Syndrome, as proposed by both Okuyama et al. and Lands et al. three decades ago (80, 81).

The potential ramifications of AI ancestry, the presence of the ancestral haplotype, and low levels of n-3 HUFA on critical cardiometabolic and inflammatory risk factors were next examined. AI ancestry is positively associated with levels of circulating triglycerides (TGs) and much of this effect was explained by variation in the *FADS* locus (39, 82–91). The high frequency of the ancestral *FADS* alleles together with their effect size in AI-Ancestry Hispanic populations suggest that *FADS* variation is particularly relevant to TG levels in this population. The liver is important in TG synthesis and deficiencies of n-3 HUFA and imbalances of n-6 relative to n-3 PUFA and HUFA have been associated with elevated TGs and non-alcoholic fatty liver disease (NAFLD) (92). A strong association between the *FADS* variant rs174537 and E-selectin was also observed in Hispanic populations, with higher levels in individuals carrying copies of the ancestral T allele (39). Circulating levels of E-Selectin (CD-62E) are elevated in many diseases involving chronic inflammation including obesity (93), cardiovascular disease (94), bronchial asthma (95), and cancer (96, 97).

The original observation by Fumagalli et al. that strong signals for the ancestral haplotype in the *FADS* cluster are associated with anthropometric characteristics such as body weight and height are curious (57). We also examined the effect of rs174537 and rs174557 on the same set of phenotypes and found them to be significantly associated with higher waist-hip circumference ratio, as well as lower height and weight. The rs174537 allele T further demonstrated an association with reduced height and weight in the large Hispanic Community Health Study/Study of Latinos (HCHS/SOL) cohort (*n* = 12,333) (39). The capacity of this region of the genome to impact anthropometric characteristics likely played a key evolutionary role in cold adaptation for early Siberian and indigenous American populations. Additionally, it may also be vital in impacting key CVD risk factors in modern AI ancestry Hispanic and Native American population. While it is unclear which signaling molecules from *FADS* derived steps are responsible for the anthropometric changes, our recent combined genetic and metabolomic analyses showed the *FADS* locus is a central control point for biologically active HUFA-containing complex lipids that act as signaling molecules. The endocannabinoid, 2-AG, and such endocannabinoids are known to impact anthropometric and other phenotypic characteristics (38).

## *Post Hoc* Subgroup Analysis of the Vital Trial

Imbalance of n-6 to n-3 HUFA could likely be attenuated by n-3 HUFA enriched diets or supplementation thereby preventing or improving serious disease outcomes; however, this relationship needs to be evaluated in the context of *FADS* genetic variants and/or ancestry. Results from randomized clinical trials with EPA and DHA (mostly with European ancestry participants) have shown conflicting results, and thus their efficacy for reducing CVD and cancer remain controversial. The 2019 Vitamin D and Omega-3 Trial (VITAL) is of particular interest here, as it included *n* = 5,106 African American participants out of *n* = 25,871 total participants. Overall, they reported that supplementation with either n−3 HUFA (EPA + DHA, provided as ethyl esters) at a dose of 1 g/day or vitamin D3 at a dose of 2,000 IU/day was ineffective for primary prevention of CVD (composite endpoint) or cancer events among their entire study cohort of healthy middle-aged men and women over 5 years of follow-up (98). However, in line with the *FADS* hypothesis outlined above, for African American participants they reported (in the supplemental material) an overall reduction in myocardial infarction of 77% (vs. placebo), and a marked reduction in coronary revascularization and total coronary heart disease with EPA and DHA supplementation.

**Figure 5:**
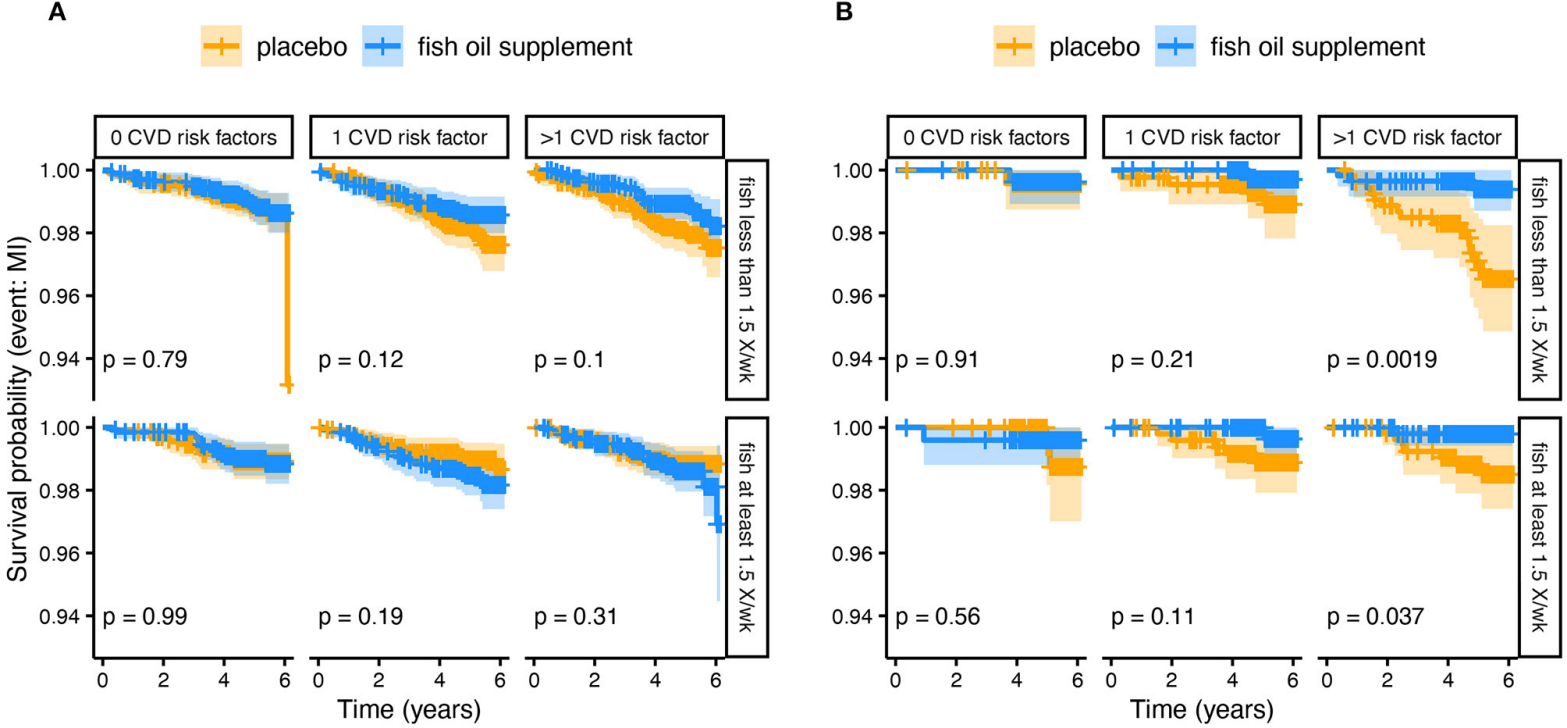
Kaplan-Meier survival curves comparing n-3 HUFA supplementation and placebo from a subgroup analysis of **(A)** White and **(B)** African American Subjects. The plots have been divided by fish intake (at least 1.5× per week) and the number of cardiovascular disease (CVD) risk factors. The plotted lines show the probability of surviving (i.e., not experiencing a myocardial infarction) over time. The lighter shaded regions surrounding the plot lines show 95% confidence regions. The greatest difference is observed in African Americans who did not eat fish and had >1 CVD risk factors, such that those receiving n-3 supplements had many fewer myocardial infarction events over the study period.

Using the VITAL study data, we performed additional subgroup comparisons based on the *FADS* framework. Figure 5 compares the Kaplan-Meier survival curves for the myocardial infarction endpoint, faceted by fish consumption and the number of cardiovascular risk factors, for both White and African American subjects. These data show the largest impact of n-3 HUFA supplementation on African American subjects who do not consume fish and have existing cardiovascular risk factors, suggesting that this group in particular could benefit from n-3 HUFA supplementation. In line with our expectations based on the mixed distribution of *FADS* haplotypes in European-ancestry populations, we do not see such a response to n-3 HUFA supplementation in the White subjects. These results come with all the usual caveats for *post-hoc* re-analyses of clinical trial data and there is always a risk of finding false positive associations. However, in this case, the pattern of subgroup results agrees with predictions based on *FADS* genetics and resulting HUFA metabolism.

## Conclusions

The human consumption of LA has risen dramatically over the past 100 years (2), due largely to manipulation of food supplies, increases in processed foods, and dietary recommendations. LA-containing food and oils are currently being consumed by highly diverse populations in countries such as the US whose populations represent numerous ancestries and evolutionary histories. Ancestry-influenced *FADS* variability together with current levels of LA in Western diets likely place certain individuals and populations at greater risk for disease due to elevated n-6 HUFA or markedly reduced n-3 HUFA levels and concomitant alterations in n-6 HUFA to n-3 HUFA ratios. These imbalances manifest themselves as alterations in levels of lipid signaling metabolites that impact inflammatory and thrombotic conditions.

Data from the studies of African ancestry populations raise concerns as to the impact of a Western diet (containing 6–8% LA and a LA to ALA ratio >10:1) combined with a high frequency of alleles associated with efficient HUFA biosynthesis. Recent clinical trials (offering empirical evidence of gene-nutrient interactions) suggest that this gene-diet interaction would lead to a marked increase in the production of ARA relative to DGLA as well as reducing n-3 HUFA (1, 37, 66, 67).

Our findings with African Americans in the VITAL trial further highlight the urgent need to consider genetic ancestry (or race as proxy) and/or *FADS* genotypes in n-3 HUFA supplementation trials and dietary recommendations. By focusing on a one-size-fits-all outcome (i.e., all subjects), the impact of *FADS* variation gets obscured, and subgroups who could benefit miss out. In this case, many African Americans who may have benefited from n-3 HUFA supplementation likely did not receive a recommendation or prescription due to reporting of the overall negative result.

This will continue as long as HUFA metabolism and n-3 HUFA supplementation are viewed through a single lens for all people. Instead, future work should expect this diversity of responses and focus on those groups for which placing preformed n-3 HUFA into the diet would be the most helpful. Ultimately, considering individual genotypes at *FADS* and other loci will likely lead to personalized supplementation and dietary recommendations.

## Author Contributions

AM, CY, MT, and SR carried out the analysis in the manuscript with the MESA data. TO'C analyzed genetic population data and created Figure 2. SB and SS help with the genetic and biochemical analyses in the manuscript and created Figures 1, 3. LJ and BH carried out the VITAL *post hoc* subgroup analyses. FC oversaw the various analyses shown in the manuscript. FC, RM, SMS, SB, BH, and AM contributed to writing the manuscript. All authors contributed to the article and approved the submitted version.

## Funding

This work was supported by NCCIH R01 AT008621 and USDA ARZT-1361680-H23-157. The Multi-Ethnic Study of Atherosclerosis (MESA) and the MESA SHARe projects are conducted and supported by the National Heart, Lung, and Blood Institute (NHLBI) in collaboration with MESA investigators. Support for MESA is provided by contracts HHSN268201500003I, N01-HC-95159, N01-HC-95160, N01-HC-95161, N01-HC-95162, N01-HC-95163, N01-HC-95164, N01-HC-95165, N01-HC-95166, N01-HC-95167, N01-HC-95168, N01-HC-95169, UL1-TR-000040, UL1-TR-001079, UL1-TR-001420, UL1-TR-001881, and DK063491. Funding for SHARe genotyping was provided by NHLBI Contract N02-HL-64278. Genotyping was performed at Affymetrix (Santa Clara, California, USA) and the Broad Institute of Harvard and MIT (Boston, Massachusetts, USA), using the Affymetrix Genome-Wide Human SNP Array 6.0.

## Conflict of Interest

FC is a co-founder of a start-up company TyrianOmega, which focuses on the production of omega-3 PUFAs by cyanobacteria, largely for animal feeds and aquaculture. The remaining authors declare that the research was conducted in the absence of any commercial or financial relationships that could be construed as a potential conflict of interest.

## Publisher's Note

All claims expressed in this article are solely those of the authors and do not necessarily represent those of their affiliated organizations, or those of the publisher, the editors and the reviewers. Any product that may be evaluated in this article, or claim that may be made by its manufacturer, is not guaranteed or endorsed by the publisher.
